# Recent advances in the management of peptic ulcer bleeding

**DOI:** 10.12688/f1000research.11286.1

**Published:** 2017-09-27

**Authors:** Ian Beales

**Affiliations:** 1 Department of Gastroenterology, Norfolk and Norwich University Hospital, Norwich, UK

**Keywords:** gastrointestinal hemorrhage, peptic ulcer hemorrhage, endoscopic hemostasis, H pylori, anticoagulant drugs, non-steroidal anti-inflammatory drugs

## Abstract

Acute upper gastrointestinal haemorrhage due to peptic ulcer bleeding remains an important cause of emergency presentation and hospital admission. Despite advances in many aspects of management, peptic ulcer bleeding is still associated with significant morbidity, mortality, and healthcare costs. Comprehensive international guidelines have been published, but advances as well as controversies continue to evolve. Important recent advances include the evidence supporting a more restrictive transfusion strategy aiming for a target haemoglobin of 70–90 g/l. Comparative studies have confirmed that the Glasgow–Blatchford score remains the most useful score for predicting the need for intervention as well as for identifying the lowest-risk patients suitable for outpatient management. New scores, including the AIMS65 and Progetto Nazionale Emorragia Digestiva score, may be more accurate in predicting mortality. Pre-endoscopy erythromycin appears to improve outcomes and is probably underused. High-dose oral proton pump inhibition (PPI) for 11 days after PPI infusion is advantageous in those with a Rockall score of 6 or more. Oral is as effective as parenteral iron at restoring haemoglobin levels after a peptic ulcer bleed and both are superior to placebo in this respect. Within endoscopic techniques, haemostatic powders and over-the-scope clips can be used when other methods have failed. A disposable Doppler probe appears to provide more accurate determination of both rebleeding risk and the success of endoscopic therapy than purely visual guidance. Non- *Helicobacter pylori*, non-aspirin/non-steroidal anti-inflammatory drug ulcers contribute an increasing percentage of bleeding peptic ulcers and are associated with a poor prognosis and high rebleeding rate. The optimal management of these ulcers remains to be determined.

## Introduction

Acute upper gastrointestinal (GI) haemorrhage remains an important clinical problem. The incidence of non-variceal acute upper GI bleeding in the UK is approximately 85 per 100,000 per year ^1^. Although the specific mortality associated with acute variceal bleeding is higher ^2^, peptic ulcer bleeding (PUB) remains the commonest cause of acute GI bleeding overall and significant bleeding requiring transfusion ^2, 3^. Despite considerable advances in many aspects of the management of PUB, the overall mortality remains significant (approximately 10%), the increasing age and comorbidity of the patients somewhat offsetting the therapeutic advances.

There are several evidence-based guidelines to aid the management of PUB ^4, 5^, although comprehensive audits have shown that all aspects of management do not always reliably follow guidelines ^3^. New information is continually becoming available in all aspects of PUB, and in many cases these have not had time to directly inform guideline development. In this review, important recent developments in all aspects of managing PUB are discussed and relevant controversies placed into context to aid the practical management of ulcer bleeding.

The management of ulcer bleeding can be divided for convenience into areas of recognition, risk assessment, resuscitation, endoscopic and salvage therapies, and drug therapies in both the peri-bleeding and post-bleeding situations.

## Recognition

Usually the presentation of acute upper GI bleeding is obvious to the clinician, certainly once the presence of blood in the vomitus or melaena passed rectally is detected. One significant dilemma remains over the likely site of bleeding for profuse, haemodynamically significant fresh rectal bleeding. Is this from a colonic source or very rapid transit from an upper GI source? This has implications for the investigative process. In the United Kingdom, nationwide review of severe GI bleeding, general clinical features, and suspicion in this situation were poorly correlated with the actual site of bleeding ^3^. The presence of a pulse rate greater than the systolic blood pressure was associated with an upper GI source for fresh rectal bleeding ^3^, and although further studies examining this index in a prospective way are required, it certainly seems reasonable to perform a gastroscopy initially before lower GI endoscopy in these patients showing that degree of circulatory compromise after appropriate resuscitation.

## Resuscitation

Despite the high prevalence of PUB, there are few data on any specifics of fluid resuscitation in this context. The general clinical principles on restoring circulating fluid volume and adequacy of organ perfusion are employed, although it seems inevitable that there will be individual choice in terms of fluids used and rate given. Obviously, randomised studies in this area are extremely difficult to do and hence the widely cited trial examining blood transfusion strategies was extremely welcome ^6^, although the limitations inherent in recruiting into such a trial in acute bleeding must always be considered when reviewing the results. In common with an increasing body of data from other critically ill patients, across the spectrum of medicine including major sepsis ^7, 8^, a restrictive blood transfusion strategy seems at least as good as a more traditional liberal strategy. In the key trial ^6^, the strategy of a single unit as required, and repeated as necessary, with a transfusion trigger of 70 g/l to maintain the haemoglobin at 70–90 g/l was as safe and effective as a more traditional haemoglobin target of 90–110 g/l. The trial recruited all comers with upper GI bleeding and was not specifically designed to look at subgroups. Overall mortality was lower in the restrictive transfusion group (5% versus 9%). Those with variceal bleeding or Child’s A or B cirrhosis particularly seemed to benefit from the conservative transfusion strategy with improved mortality and rebleeding rates. In those with PUB, there was less difference between the two strategies, although all important clinical outcomes favoured the restrictive strategy (mortality 3% versus 5%, rebleeding 10% versus 17%, and surgery 2% versus 6%). Recruitment to the trial was rather selective, excluding those with very severe bleeding and significant circulatory diseases, which may limit generalisation. Data published only in abstract form ^9^ examining real-world outcomes after transfusion for PUB suggested a contrary finding and showed an association between more units transfused and lower death rates.

A meta-analysis pooled the results from the four available studies of transfusion strategies in acute upper GI bleeding, although all have different methodologies and inclusion criteria ^10^. Not all of these included studies specifically examined only acutely bleeding peptic ulceration. Again, results favoured a restrictive strategy: there were significant reductions in death and length of stay with the restrictive strategy. Rebleeding rates were also non-significantly lower in the restrictive group (odds ratio 0.26, 95% confidence interval [CI] 0.03–2.10). An even more recent meta-analysis including only data on acute GI bleeding from five randomised controlled trials showed that a restrictive transfusion strategy was associated with lower all-cause mortality (relative risk 0.65, 95% CI 0.44–0.97) and rebleeding (relative risk 0.58, 95% CI 0.40–0.84) without any effect on ischaemic events ^11^. The exact optimal resuscitation strategy is unclear and always needs to be individualised to the specific patient. However, given the consistency and biological plausibility of the results and the costs and potential harm of blood transfusion, it would seem prudent to employ a conservative transfusion strategy for most patients with PUB, whilst maintaining adequate circulating fluid volumes.

It is important to stress that transfusion strategies are but one part of fluid resuscitation in PUB and that in the acutely bleeding patient haemoglobin levels form only one part of the assessment of cardiovascular instability and that decisions on fluid and blood replacement must be governed by the need to restore adequate organ perfusion. However, it does seem that crystalloid fluid resuscitation followed by blood to maintain the haemoglobin level at 70–90 g/l is most appropriate for most patients; further data are required for those with severe or critical vascular and circulatory diseases.

## Risk stratification

There are many systems that have been used to stratify risks in upper GI bleeding. Probably the two most widely used and studied are the Rockall scores (both pre- and post-endoscopy) and the Glasgow–Blatchford score (GBS) ^12^. Although these have always been designed to assess somewhat different aspects, there continue to be studies comparing the clinical utility of these studies. It must be remembered that the Rockall scores assess mortality risk but were never designed directly as decision tools (accepting that the risk assessment of the patient clearly does inform clinical decision-making indirectly) but that the GBS was explicitly designed and validated to predict those cases not needing intervention (therapeutic endoscopy or blood transfusion). Thus, not surprisingly, the GBS consistently performs better in identifying lower-risk cases, suitable for direct discharge and outpatient management ^13, 14^. Further recent international validation of the GBS has confirmed that a score of 0 or 1 is associated with a very low risk of intervention and that hospital admission and emergency endoscopy are not required ^15^.

Further scores have been proposed. The AIMS65 score has been advocated as an even simpler score requiring scoring only on a 5-point score for each of the following factors: albumin of less than 30 g/l, international normalised ratio (>1.5), Glasgow coma scale score of less than 14, systolic blood pressure of less than 90 mmHg, and age of more than 65. Although the AIMS65 can reliably predict mortality, it appears less accurate than the GBS in determining the need for interventions such as blood transfusion or admission to critical care ^15, 16^.

A further score, the Progetto Nazionale Emorragia Digestiva (PNED) score system, which relies on a rather complex multipart scoring using age, presence of cancer, renal failure, American Society of Anaesthesiologists grade, cirrhosis, rebleeding, and failure of endoscopic therapy, has been proposed. A large prospective study of over 3,000 patients confirmed that the GBS clearly performed best in identifying the lowest-risk patients and also in predicting interventions such as blood transfusion or endoscopic therapy. Although the PNED and AIMS65 scores were best at predicting mortality, none of the scores apart from the GBS appeared to be clinically useful in determining either the safety of outpatient management or the need for endoscopic therapy ^15^. A GBS of 7 or more was best at predicting the need for endoscopic treatment ^15^.

A further score based on seven factors—systolic blood pressure of less than 100 mmHg, syncope, haematemesis, haemoglobin of less than 100 g/l, blood urea of 22.4 mg/dl, estimated glomerular filtration rate of less than 60 mL/min per 1.73 m ^2^, and the use of anti-platelet medications—was recently proposed ^17^. This score was superior to the pre-endoscopy Rockall and AIMS65 scores in predicting clinical intervention in a cohort of Japanese patients ^17^ but has not been compared against the GBS or evaluated more widely.

Timing of emergency endoscopy in acute upper GI bleeding remains a controversial area, and although immediate endoscopy (as early as possible) seems theoretically attractive, this has not been supported by evidence. Studies have shown that very early endoscopy is not associated with better outcomes and in some cases is associated with worse outcomes (although this latter effect could have been an artefact of the design of the observational studies) ^18, 19^. More recently, those patients with a GBS of 12 or more were shown (again in an observational study) to have lower mortality with a presentation to endoscopy time of more than 13 hours, whereas those with lower GBSs did not seem to benefit from such early endoscopy ^20^. This suggests that GBSs can be used both to triage patients not needing admission and to detect those who may benefit from relatively early endoscopy.

Further modifications of the GBS have also been reported, removing the most subjective of the criteria and relying merely on measurable haemodynamics and laboratory values, omitting the scoring for chronic disease/major comorbidities, melaena, and syncope. Interestingly, the abbreviated score seemed to perform as well as the full GBS and was again superior to the Rockall scores at predicting the need for clinical intervention ^21^. Whilst further validation studies are required, this may prove to be a useful modification in clinical practice.

## Endoscopy and endoscopic therapy

Dual therapy, that is adrenaline/epinephrine infiltration plus either thermal coagulation with a bipolar probe or mechanical haemostasis with endoclips, remains the optimal endoscopic therapy advocated in major guidelines ^4, 5^. However, the thermal or mechanical aspects are the most important, and although adrenaline is often used to clear the endoscopic field, it probably adds little to the haemostasis as secured by these other means ^12^. Although there are a variety of through-the-scope endoscopic clips available, there are no data showing clear superiority of any one type.

Within endoscopy, there are three important recent developments: Doppler probe-guided lesion assessment and treatment, large over-the-scope clips, and haemostatic powders. The exact place of all of these within the management pathway requires further assessment, but all seem to offer some advantages in certain circumstances.

Doppler probe assessment to detect significant arterial signals in the ulcer base had been reported many years previously ^22^. However, the lack of availability of the equipment and lack of convincing evidence of efficacy at the time rather precluded further adoption. More recently, there has been an increase in interest, stimulated by the availability of an easier-to-use Doppler unit and disposable, relatively low-cost endoscopic probes (Vascular Technologies Inc., Nashua, NH, USA). Two studies from the same group have shown initially how Doppler probe assessment is more accurate than classic endoscopic scoring at predicting rebleeding risks ^23^ and secondly, in a randomised trial, that Doppler probe-guided management reduces rebleeding and further intervention compared with standard treatment ^24^. Doppler assessment showed that many oozing ulcers (Forrest 1b) are actually not associated with significant arterial flow into the ulcer (only 46.7% showed a positive Doppler signal) and that these ulcers are associated with a lower rebleeding rate than typically assumed. The rate of Doppler-positive arterial flow in oozing ulcers is actually significantly lower than the prevalence of positive Doppler signals for active arterial bleeding (100%), non-bleeding visible vessel (Forrest 2a, 90.7%), and those with adherent clot (Forrest 2b, 68.4%). Interestingly, 40.5% of ulcers with flat haem spots alone (Forrest 2c), which are classically associated with a low risk of rebleeding, had a positive arterial Doppler signal, whilst in clean-based ulcers (Forrest 3) only 8.3% had a positive Doppler signal. Repeating Doppler assessment post-standard endoscopic treatment showed a considerable reduction in arterial flow, and persistent arterial inflow was associated with an increased risk of rebleeding ^23^.

A subsequent randomised trial compared the use of the Doppler probe to inform both the indication for therapy and the success of that therapy against standard haemostatic treatment based purely on endoscopic visualisation ^24^. Endoscopic therapy was applied on the basis of the presence of a Doppler signal rather than endoscopic appearance, and after endoscopic therapy, the ulcer was re-interrogated and retreated if an arterial signal was still present. The Doppler probe allows clearer localisation of the feeding artery. Overall, Doppler use in this manner was associated with a significant reduction in rebleeding. Rates of rebleeding at 30 days were 8/72 (11.1%) in the Doppler-treated group and 20/76 (26.3%) in the standard care group. Residual arterial signal despite maximal endoscopic therapy (adrenaline, bipolar probe, through-the-scope clips) was strongly associated with rebleeding (8/9 cases, 88.9%) compared with 0/8 (0%) of those who had continued endoscopic retreatment until the Doppler signal was obliterated.

This technique looks very promising. The application of the Doppler probe allows more accurate definition of the rebleeding risk of ulcers (superior to standard endoscopic stigmata), facilitates tracing of the underlying artery for direction of haemostatic methods, and allows post-treatment interrogation to define the efficacy of endoscopic therapy. Further studies in other populations with less experienced and committed operators are required before widespread adoption, and further data on the efficacy of this advance are awaited with interest.

These data with the Doppler probe showing that oozing ulcers (Forrest 1b) are associated with significantly lower risks of rebleeding post-endoscopic therapy ^23, 24^ are in keeping with a retrospective analysis of data from one of the large studies of proton pump inhibitor (PPI) therapy after endoscopic therapy ^25^. In the placebo-treated group, rebleeding was much lower in those with oozing ulcers (4.9%) than in those with spurting (Forrest 1a, 22.5%), adherent clot (Forrest 2b, 17.6%), and visible vessel (Forrest 2a, 11.3%). It was previously thought that as oozing ulcers were seen to be ‘actively bleeding’ that these were high-risk lesions. These recent data confirm that Forrest 1b lesions tend to have smaller feeding arteries and hence rebleeding rates are lower than previously believed. Interestingly, this study also showed that intravenous esomeprazole did not reduce the already-low post-endoscopic rebleeding rate in the oozing ulcers (in comparison with the other high-risk stigmata), suggesting that parenteral acid suppression may be withheld from this group after successful haemostasis and standard oral therapy used ^25^. This reappraisal of rebleeding rates associated with classic endoscopic stigmata of recent haemorrhage has important implications for the interpretation of existing studies and the design of future studies as grouping all active bleeding groups (Forrest 1a and 1b) together now seems inappropriate given the clearly divergent rebleeding risks.

The main limitations of typical endoscopic clips are their relatively small size and the pressure that the jaw can apply to close tissue or provide mechanical haemostasis. The much larger and stronger over-the-scope endoscopic clip (OTSC, Ovesco Endoscopy, Tubingen, Germany) overcomes many of these drawbacks, being able to grasp larger and more fibrotic areas than standard clips and to apply more pressure onto feeding arteries. The obvious cost of this is having to preload the clip on the endoscope before intubation and much greater unit cost and sometimes difficulty passing the clip through the upper oesophageal sphincter. The OTSC clip is US Food and Drug Administration (FDA)-approved and available in many areas, being utilised to close fistulas and perforations in addition to acute PUB. Several case series have reported successful haemostasis with this device when other endoscopic methods have failed. Honegger *et al*. ^26^ reported 85% success in treating PUB (28/35), although in a smaller case series, haemostatic success was reported in only 4/7 cases of refractory bleeding peptic ulcer ^27^. The OTSC clip has also been used successfully as primary treatment for PUB: Manno *et al*. reported 100% success in 21 cases ^28^. There are no randomised or indeed comparative studies available at present, but as a second-line endoscopic technique these clips seem to provide a further useful tool.

Haemostatic powders are in a similar position. These seem to be a promising technology but are not yet supported by comprehensive randomised trial data. There are now several powders commercially promoted in various geographical locations, the first being Hemospray (Cook Medical, Bloomington, IN, USA) but others are now available, although they are not yet FDA-approved for use in the USA. These are proprietary mineral preparations that, when sprayed onto a bleeding area through a cannula inserted through the channel of an endoscope, provoke rapid haemostasis. The powder acts as both a physical barrier upon contact with moisture and a powerful procoagulant by concentrating clotting factors at the site of application. Again, there are no randomised trials, but several case series showing successful haemostasis after failure of first-line endoscopic therapies show that this technique can also be usefully employed in the most difficult refractory bleeding ulcers. In a comprehensive literature review of reported cases, Hemospray was successful in 88% of 81 cases of bleeding peptic ulcers ^29^. Obviously, this method provides no destruction of the underlying artery (as clips or bipolar probes do), and the rate of rebleeding and the subsequent natural history of PUB bleeding in this manner are unknown. Haemostatic powders do not influence the underlying arterial inflow, and at present it is unclear whether rebleeding rates with highest-risk stigmata (spurting arteries or those with significant positive Doppler traces) are clinically problematical. The powder application invariably obscures the endoscopic view, and perhaps repeat second-look endoscopy will be required to perform more secure haemostasis. At present, this cannot be regarded as a routine first-line therapy but in some cases can be extremely useful when other methods have failed. The technique is relatively easy, although care must be taken to avoid premature exposure of the powder to liquid, which activates the powder. In the author’s experience, applying this in a duodenal cap with a rapidly bleeding artery is often quite difficult but can be applied for bleeding lesions when other methods are technically impossible or have failed and may provide rescue haemostasis in those cases. It is important to note that the requirement for blood or liquid for effective activation often precludes the use of haemostatic powders on non-bleeding but protuberant arteries (Forrest 2a lesions) that do merit some form of endoscopic therapy. Further data reporting the different haemostatic powders in relation to more standard haemostatic methods and in ulcers with different bleeding stigmata will help refine the place of the powders in management.

## Drug therapy

Pre-endoscopy PPI infusion is recommended by some guidelines but not by all ^12^. Although this seems to downstage the endoscopic appearance of bleeding ulcers, the effect on hard clinical end-points such as rebleeding or hospital stay is debateable.

Post-endoscopy PPI treatment after endoscopic therapy to high-risk ulcers has repeatedly been shown to be better than placebo at reducing rebleeding and surgery ^30^. However, despite a multitude of studies, the optimal regimen is unclear. Many clinicians use the original ‘Hong Kong’ regimen (bolus followed by continuous infusion of omeprazole, pantoprazole, or esomeprazole) for 72 hours. Other dose regimens, including intermittent parenteral dosing and even high-dose oral PPI, have also been shown to be effective, and it is not clear what the optimal regimen is ^31, 32^. As previously discussed, the rebleeding rate after successful endoscopic haemostasis in oozing (Forrest 1b) ulcers is low and does not seem to be reduced by parenteral high-dose acid suppression and hence treatment may be rationalised in those patients to standard oral PPI therapy ^25^.

After endoscopic therapy and 72 hours intravenous PPI, high-dose oral acid suppression seems to be beneficial for highest-risk patients. Cheng *et al*. ^33^ reported that 11 days of double-dose oral esomeprazole (40 mg twice daily) in this context was superior to once-daily esomeprazole 40 mg (with 40 mg once daily subsequently for both groups) in preventing rebleeding (10.8% versus 28.7% in the 4–28 days post-index bleed) in patients with a full Rockall score of 6 or more. There was no significant difference in mortality, hospital stay, or blood transfused. Thus, there is a rationale for treating the higher-risk patients (Rockall score of 6 or more) with higher-dose PPI for the period after initial stabilisation.

There is a sound rationale for using prokinetics before endoscopy in upper GI bleeding to clear the stomach and improve both the endoscopic views and probably safety. Individual trials have shown inconsistent results, but recent meta-analyses showed that intravenous erythromycin before endoscopy was associated with meaningful clinical benefit in terms of improved mucosal visualisation, reduction in repeat endoscopy, and blood transfused as well as length of stay but that metoclopramide was less effective ^34– 36^. Erythromycin is probably underused and seems to be a simple intervention that would improve outcomes.

The management of concurrent anticoagulation is an increasing problem for those involved in the care of acute PUB. The use of prothrombin complex concentrate to reverse the anticoagulation effects of vitamin K antagonists (VKAs) such as warfarin is well established ^12^. The direct-acting oral anticoagulants (DOACs), the thrombin antagonist dabigatran, and the factor X inhibitors apixaban, rivaroxaban, and edoxaban present more of a problem. Although the anticoagulation effect declines relatively rapidly because of renal clearance, life-threatening bleeding will require reversal in some patients. The first specific reversal agent for dabigatran has just been licenced and, though expensive, should be available to treat significant dabigatran-associated PUB. Idarucizumab is a monoclonal antibody against dabigatran and will not reverse the other DOACs ^37, 38^. There are relatively few data on treatment of upper GI bleeding associated with these agents, although widespread use of tranexamic acid is not routinely indicated in PUB ^39^ (although the results of the large worldwide HALT-IT trial are awaited with interest ^40^); in this particular situation, the use of tranexamic acid seems reasonable ^41^. Prothrombin complex concentrate seems to reverse the anticoagulation effect of factor X inhibitors in healthy volunteers and should probably be considered in severe life-threatening bleeding, although there are really no data specifically showing an effect in PUB ^42^. A specific antidote to factor Xa inhibition has been shown to rapidly reverse the anticoagulant effect and hopefully will be available for clinical use soon ^43^.

Acute upper GI bleeding is a significant drain on the iron stores of the body, and many patients are anaemic after initial management. A randomised trial compared subsequent management strategies in this group in non-variceal upper GI bleeding (mostly peptic ulcer-related). Iron therapy—either a one-off dose of ferric carboxymaltose or oral ferrous sulphate 200 mg daily—was more effective than placebo at restoring haemoglobin levels to normal. After 12 weeks, 70% of placebo-treated and 17% of iron-treated patients were still anaemic ^44^. There was no difference in the rates of improvement in anaemia between parenteral and enteral iron groups, although higher ferritin levels were seen in the parenteral group ^44^. There was no significant toxicity, and it seems logical that supplemental iron therapy should be used in those patients with anaemia at the cessation of the peptic ulcer bleed.

## Management of refractory bleeding

Despite advances in endoscopic and pharmacological therapies, a significant minority of patients experience significant rebleeding. Surgery has traditionally been regarded as the appropriate approach. Increasingly, interventional radiology is regarded as the initial therapeutic approach before surgery. There are no randomised trials to guide therapy, nor are there likely to be. Though not supported by trial data, the author’s own practice is to place endoclips to mark the site of bleeding in those deemed at highest risk of needing embolization (most obviously in those with failed endoscopic haemostasis but also in those with technically difficult but successful haemostasis) to facilitate subsequent radiological localisation.

A number of case series have reported high technical success and acceptable complication rates with radiological embolization for acute PUB ^45– 47^, and it is believed that overall the safety of interventional radiological embolization is significantly better than surgery and hence most guidelines now advocate radiological embolization as the rescue therapy of choice. However, not all studies agree: single-centre observational studies and a meta-analysis have suggested that rates of rebleeding are higher following embolization and mortality rates equivalent ^48, 49^. All such studies are somewhat difficult to interpret because of case mix: patients undergoing embolization tended to be older and have more comorbidities.

## Follow-up and prevention

An understanding of the major causes of PUB naturally leads to developing strategies for both primary and secondary prevention. The major ameliorable causes of PUB are *Helicobacter pylori* and drugs, although it is important to note the apparent rise in idiopathic—non- *H. pylori*, non-aspirin/non-steroidal anti-inflammatory drug (NSAID)—ulcers.

Strategies to deal with *H. pylori* need to encompass both the declining efficacy of eradication therapies and the poor sensitivity of *H. pylori* testing in the context of PUB ^50^. Empirical eradication at the presentation with bleeding has been advocated in areas with a high prevalence of *H. pylori*, whilst an alternative approach of careful follow-up testing and focused treatment may be more applicable in areas with a low prevalence of *H. pylori*-induced ulcers ^51^. In any case, *H. pylori* eradication regimes must be effective in the population being treated and 14-day courses of four agents (either bismuth-containing or not) are now standard in Europe and the USA, although 7-day clarithromycin-containing regimens are still used in the UK in areas with a known low prevalence of clarithromycin-resistant *H. pylori* (for a full review of *H. pylori* eradication strategies, see the Maastricht V guidelines ^52^). It is essential that all those treating *H. pylori* are alert to the efficacy of their current treatment regimens and follow up patients assiduously.

For many years, it has been established that the sensitivity of all endoscopy-based *H. pylori* tests is lower in acute PUB ^50^. The reasons for this are unclear and are not as simple as being affected by blood in the lumen. The yield of biopsy-based tests can be significantly improved by taking additional biopsies from the gastric body ^50^, but owing to a residual false-negative rate, careful follow-up testing may still be more appropriate in those initially negative ^51^. In the acute bleeding setting, ^13^C urea breath testing on return from endoscopy seems to be the most accurate test for *H. pylori* but many units find this logistically difficult to organise ^50^. In contrast, the faecal antigen test has a high false-positive rate in acute PUB, possibly due to cross-reaction with blood components in the GI lumen, and cannot be recommended for *H. pylori* testing in the acute setting ^50^. The diagnostic yield for *H. pylori* in the context of PUB can be significantly enhanced by performing diagnostic testing at least 4 weeks after the index bleed ^53^.

Although aspirin and other anti-platelet agents are clearly associated with an increased risk of PUB, in many cases these agents are indicated because of the underlying vascular disease, and it is now accepted that where indicated aspirin should be continued (or interrupted for a minimal interval of fewer than 3 days) in acute PUB ^51^. A small risk in early rebleeding is more than offset by a significantly reduced risk of vascular events and death. This approach is supported by data from both a relatively small clinical trial and observational follow-up data ^54, 55^.

The most appropriate treatment after an aspirin-induced bleed is aspirin plus a PPI ^56^; this is superior to the P _2_Y _12_ antagonist clopidogrel alone as secondary treatment. There are fewer data on the newer P _2_Y _12_ antagonists ticagrelor and prasugrel, but these are more potent anti-platelet agents, and not surprisingly the risk of GI bleeding seems to be higher than aspirin or clopidogrel ^57, 58^. Patients with drug-eluting coronary artery stents do need to continue dual anti-platelet therapy for a year; PPI co-treatment reduces bleeding in those taking aspirin plus clopidogrel ^59^. The relative benefits and risks of aspirin plus PPI versus clopidogrel plus PPI after an aspirin-induced PUB are unclear. Observational data suggest that aspirin is safer, more effective, and preferable ^60, 61^. An interesting effect of the increased use of gastro-protection with anti-platelet agents is that as the incidence of upper GI bleeding has decreased, the incidence of lower GI bleeding has remained stable, such that in patients taking dual anti-platelet agents with PPI cover, the risk of lower GI bleeding is now approximately three times higher than that of upper GI bleeding ^62^.

In general, PPI co-treatment has been advocated with aspirin for primary and secondary prevention ^51^, although a recent study looking at secondary prevention in a Chinese population showed that famotidine was equivalent to rabeprazole ^63^. Previous data suggested that PPI treatment was better ^64^, and until more data in wider populations are available, PPI treatment remains the treatment of choice.

Similar to the case with anti-platelet agents, it is now becoming apparent that where indicated early resumption of anticoagulation for atrial fibrillation after a PUB is beneficial; again, a risk of rebleeding is more than offset by reductions in stroke and death. The exact optimal time for reintroduction of anticoagulation after a PUB is unclear; leaving reintroduction for 3 months imposes an excessive risk of thrombotic events ^65, 66^, whilst very early reintroduction does increase the rebleeding risk, and the compromise of reintroduction after 7–15 days seems to provide the optimal reduction in thromboembolic events compared with rebleeding ^67, 68^. More studies specifically examining this effect are required.

Previous studies have confirmed that selective COX-2 inhibitors are safer than traditional non-selective NSAIDs in terms of GI complications ^51^. The combination of celecoxib plus a PPI is associated with the lowest risk of rebleeding after an NSAID-induced PUB, when reintroduction of anti-inflammatory therapy is required ^51^. However, concerns about the cardiovascular safety of COX-2 inhibitors led to many clinicians being reluctant to prescribe these. More recent data suggest that the increased cardiovascular risk is common to all cyclo-oxygenase inhibitors, and a large randomised study powered to look at cardiovascular rather than GI outcomes showed no inferiority of celecoxib compared with either naproxen or ibuprofen: overall, cardiovascular adverse outcome rates were comparable but celecoxib was associated with a lower incidence of GI side effects ^69^. This suggests that when really necessary celecoxib (in a dose up to 200 mg daily) plus a full-dose PPI should not be withheld from patients requiring treatment after an NSAID-induced bleed.

PPI co-treatment would be usual after a PUB, and primary prevention of PUB in higher-risk patients taking anticoagulants is usually advocated by some but not all guidelines ^51, 70– 73^. Surprisingly, there are little data to specifically support this practice; one recent study showed that omeprazole co-treatment reduced rebleeding in warfarin-treated patients, although this effect was significant only in those also taking anti-platelet drugs or NSAIDs ^74^. A further observational study showed that concurrent use of PPIs or H2-receptor antagonists were both associated with a reduced risk of acute upper GI bleeding and this effect was most marked in those with a history of peptic ulcer disease ^75^. Therefore, despite the relative lack of evidence, co-prescription of gastroprotection with anticoagulant therapy would seem to be indicated in secondary prevention; for primary prevention, a case- and risk-based approach seems sensible pending further data. Those with highest risk of bleeding are most likely to gain from the use of acid suppression.

The rate of GI bleeding with DOACs compared with VKAs remains controversial. Whilst overall bleeding rates at all sites with DOACs do seem to be lower, this is mainly driven by a reduction in cerebral bleeding and the rate of GI bleeding may actually be increased but certainly does not seem convincingly lower. Several studies have shown either a higher rate of GI bleeding with DOACs or equivalent rates to VKAs ^76– 79^. The increased GI bleeding risk of DOACs seems especially marked in the most elderly (older than 76 years).

This effect on GI bleeding may be expected, as most of the DOACs are taken as active drugs (in contrast to VKAs) and relatively higher levels and local anticoagulant effects may be seen at the level of the GI mucosa. The choice of which anticoagulant (if any) to reintroduce after a PUB currently needs to be made on an individual basis, taking into account underlying risks and comorbidity, availability of antidotes, and patient preferences. It is clear that scoring systems for thrombotic risks in atrial fibrillation (CHADS _2_-Vasc) and bleeding risk (HAS-BLED) can give important guidance on the relative risks of these important outcomes ^80^; however, there are no data specifically related to how using these scores prospectively to inform management decisions influences outcomes.

Studies from disparate geographical regions have shown an important increase in apparently idiopathic peptic ulcers as the cause of upper GI bleeding. Although this was initially reported in the Far East, studies from the UK and Europe have provided support, and these ulcers can contribute up to 40% of ulcers in some series and an even higher proportion of bleeding peptic ulcers occurring in hospital inpatients ^81– 84^. Though labelled idiopathic, they are typically seen in a more elderly population with significant comorbidity and may represent a marker of systemic vascular pathology. The importance of recognising this group lies not only in allowing secondary preventative management of PUB by cause ( *H. pylori*, drug, idiopathic) but also in appreciating that this group is associated with not only a significantly higher risk of rebleeding compared with other causes (up to 42% at 7 years) but also much higher all-cause mortality (presumably due to the associated comorbidity; 87% mortality at 7 years ^85^). Although continued acid suppression with a PPI is the logical intervention for this idiopathic ulcer group, one important study (albeit observational and not randomised) showed that acid suppression did not alter rebleeding or mortality in this group ^86^. Other approaches, perhaps with alternative mucosal protectant agents such as misoprostol, would seem to be warranted.

## Conclusions

Acute PUB remains an important clinical problem, but the management continues to be refined. Important recent developments that can be incorporated into practice include the confirmation of the usefulness of the GBS in determining the lowest-risk patients, who may be safely managed as outpatients. The GBS is most useful for predicting the need for intervention, but another score—either the Rockall or the AIM65 score—should also be assessed as a predictor of mortality to aid assessment of outcomes.

A restrictive transfusion threshold with a trigger of 70 g/l and a target of 70–90 g/l is appropriate for most patients, although transfusion triggers need to be interpreted within the context of resuscitation of the individual patient. Pre-endoscopy erythromycin is probably underused, and recent evidence supports wider use to improve endoscopic views. The addition of high-dose oral esomeprazole after initial proton pump therapy in high-risk cases (Rockall scores of 6 and above) seems to have an additional benefit in reducing rebleeding. For endoscopic treatment, the haemostatic powders and over-the-scope clips are useful tools when standard modalities are ineffective or impractical. The new Doppler equipment and disposable endoscopic Doppler probe appear to be extremely promising in more accurately predicting ulcer rebleeding and the success of endoscopic therapy and are likely to be widely used if further studies are confirmatory.

Strategies to manage the bleeding episode and prevent rebleeding need to include the management of comorbidities and risks and at present must include the management of bleeding associated with all anti-thrombotic agents, but particularly the DOACs. Specific antidotes are being developed and are just entering the clinical arena. Recent data suggest that the cardiovascular risk of celecoxib is not excessive compared with standard NSAIDs, and when used appropriately, celecoxib should once again be a useful tool in the primary and secondary prevention of PUB. Non- *H. pylori*, non-NSAID ulcers are becoming an increasing problem with a poor prognosis, and further studies are urgently required to define the safest and most effective management.
